# TRPV1-Like Immunoreactivity in the Human Locus K, a Distinct Subregion of the Cuneate Nucleus

**DOI:** 10.3390/cells7070072

**Published:** 2018-07-08

**Authors:** Marina Del Fiacco, Maria Pina Serra, Marianna Boi, Laura Poddighe, Roberto Demontis, Antonio Carai, Marina Quartu

**Affiliations:** 1 Department of Biomedical Sciences, University of Cagliari, Cittadella Universitaria di Monserrato, 09042 Monserrato (CA), Italy; marina.delfiacco@gmail.com (M.D.F.); mpserra@unica.it (M.P.S.); marianna.boi@unica.it (M.B.); laura.poddighe@gmail.com (L.P.); 2 Department of Medical Sciences and Public Health, University of Cagliari, Cittadella Universitaria di Monserrato, 09042 Monserrato (CA), Italy; demrob@unica.it (R.D.); acarai@medicina.unica.it (A.C.)

**Keywords:** human medulla oblongata, cuneate nucleus, dorsal column nuclei, TRPV1, calcitonin gene-related peptide, substance P

## Abstract

The presence of transient receptor potential vanilloid type-1 receptor (TRPV1)-like immunoreactivity (LI), in the form of nerve fibres and terminals, is shown in a set of discrete gray matter subregions placed in the territory of the human cuneate nucleus. We showed previously that those subregions share neurochemical and structural features with the protopathic nuclei and, after the ancient name of our town, collectively call them Locus Karalis, and briefly Locus K. TRPV1-LI in the Locus K is codistributed, though not perfectly overlapped, with that of the neuropeptides calcitonin gene-related peptide and substance P, the topography of the elements immunoreactive to the three markers, in relation to each other, reflecting that previously described in the caudal spinal trigeminal nucleus. Myelin stainings show that myelinated fibres, abundant in the cuneate, gracile and trigeminal magnocellular nuclei, are scarce in the Locus K as in the trigeminal substantia gelatinosa. Morphometric analysis shows that cell size and density of Locus K neurons are consistent with those of the trigeminal substantia gelatinosa and significantly different from those of the magnocellular trigeminal, solitary and dorsal column nuclei. We propose that Locus K is a special component of the human dorsal column nuclei. Its functional role remains to be determined, but TRPV1 appears to play a part in it.

## 1. Introduction

The transient receptor potential vanilloid type-1 receptor (TRPV1) is a polymodal ion channel expressed in primary sensory neurons, critically involved in the perception of mechanical and thermal stimuli as well as in pain modulation, and in allodynia and hyperalgesia in neuropathic pain [1,2,3,4,5,6,7]. Temperature (over 42 °C), low extracellular pH and capsaicin represent TRPV1 activators often used in experimental studies. To date, TRPV1 is viewed as a molecular integrator of different stimuli in the peripheral polymodal nociceptors; thus, it is activated by noxious heat, acidic and basic pH, voltage, endogenous lipid-derived compounds, and a variety of substances, among which the agonist resiniferatoxin is the best known [1,2,4,8,9,10,11]. In rodents, TRPV1 is expressed by a subset of peripheral sensory neurons involved in pain sensation [12,13,14,15,16,17,18,19,20,21,22]. Available studies on human tissue show the occurrence of TRPV1 in neurons of dorsal root ganglia (DRG) [15,23,24,25,26,27,28] and trigeminal ganglion (TG) [29,30], and their central and peripheral endings [25,31,32,33]. TRPV1, in addition to Calcitonin Gene-Related Peptide (CGRP) and Substance P (SP), is localized in primary sensory neurons and, in particular, in those of small and medium size, with poorly myelinated or unmyelinated small calibre fibres, responsible for the reception of nociceptive protopathic stimuli. It has been shown that, in primary sensory neurons, TRPV1 activation triggers the release of CGRP and SP [11,33,34,35,36,37], typical markers of the capsaicin-sensitive sensory neurons [8]. The neuropeptides in turn activate their effector cell receptors, leading to neurogenic inflammation and sensitization of nociceptors [8,11]. The aberrant activation of TRPV1 has been implicated in different neuropathological conditions including inflammation [38,39,40,41,42,43], neuropathic pain [26,27,31,43], visceral pain [40,41,43,44,45], nerve injury [43,46,47] and migraine [33]. In humans, the local injection of capsaicin has been shown to cause sensitization of the cutaneous afferents [48,49,50], release of CGRP from peripheral nerve endings [11,51,52] and pain in the deep somatic tissues [53,54,55,56].

Classically, the dorsal column nuclear complex consists of the larger cuneate, gracile and external cuneate nuclei, and of the smaller medial and lateral pericuneate nuclei, nucleus Z and nucleus X of Pompeiano and Brodal [57]. For the most part, they receive large myelinated primary afferent fibres conveying somatic epicritic, kinesthetic and proprioceptive sensation from the trunk and limbs, and relay to the thalamus and cerebellum. Several substances have been identified as synaptic neurotransmitters in the dorsal column nuclei. Thus, glutamate and glycine and gamma-aminobutyric acid act as excitatory and inhibitory neurotransmitters [58,59,60,61,62,63,64,65,66], and other molecules, such as adenosine triphosphate, acetylcholine, and monoamines, may also function as transmitters and/or modulators [58,67,68,69]. As a general rule, neuropeptide immunoreactive elements are less abundant in dorsal column nuclei than in regions that relay protopathic and nociceptive stimuli, namely the spinal dorsal horn and the spinal trigeminal and solitary nuclei, both in humans [70,71,72,73,74,75] and in laboratory animals [76,77,78,79,80,81,82,83,84,85,86,87,88,89,90,91]. Recently, we have formally defined additional distinctive subdivisions of the human dorsal column nuclei, evident from prenatal to old life [92]. Extending early observations on the presence of gray matter areas that are strongly immunoreactive to SP in the territory of the human cuneate nucleus and adjacent fascicle [70,71], we have shown that the cuneate nucleus fields rich in SP also host neural structures immunoreactive to the neuropeptides CGRP, methionine- and leucine-enkephalin, peptide histidine-isoleucine, somatostatin and galanin, the trophin glial cell line-derived neurotrophic factor, and the neuroplasticity proteins polysialylated neural cell adhesion molecule and growth-associated protein-43 [92] and references therein. Moreover, the topographical distribution of the structures immunoreactive to all those markers in relation to each other clearly showed that the neurochemistry of those cuneate nucleus gray matter fields, at variance with the remaining nuclear territory, was strikingly similar to that of the spinal cord dorsal horn and the spinal nucleus of the trigeminal nerve [70,71,92,93,94,95,96,97]. As a tribute to the place where M.D.F. first observed and described those discrete cuneate nucleus subregions, after the ancient name of our town, Cagliari, we collectively call them Locus Karalis and briefly Locus K [98,99].

With the aim of further describing the capsaicin-sensitive component of the human nervous system, here we show that the Locus K, identified by its immunoreactivity to SP and CGRP, also contains TRPV1-like immunoreactivity (LI) in specimens from prenatal and neonatal life to adult age. Furthermore, we show that the cyto- and myeloarchitecture of the Locus K also harmonize with those of the protopathic and nociceptive sensory nuclei.

## 2. Materials and Methods

### 2.1. Tissue Sampling

Specimens of medulla oblongata were obtained at autopsy from subjects with no signs of neuropathology, at age ranging 21 gestation weeks to 88 years (Table 1). The sampling and handling of human specimens conformed to the guidelines of the local Ethics Committee of the National Health System and complied with the principles enunciated in the Declaration of Helsinki. R.D. and A.C. collected the human tissues and R.D. was the only one to have access to identifying information about the autopsied subjects. The used specimens had been stored as part of the standardized procedure for autopsy samples at the section of Forensic Medicine of the Department of Public Health, Clinical and Molecular Medicine. The Ethics Committee formally stated that the present study complied with the ethical principles and does not need approval because all the used specimens were processed anonymously (Report No. 9, 15/07/2015). Fixation in 4%, freshly prepared phosphate-buffered formaldehyde, pH 7.3, for 4–6 h at 4 °C, was followed by overnight rinsing in 0.1 M phosphate buffer (PB), pH 7.3, containing 5–20% sucrose.

### 2.2. Immunohistochemistry and Histology

Adjacent transverse slices of the medulla oblongata were cut with a cryostat at 10–14 or 30 µm and collected in series on chrome alum-gelatin coated slides. The avidin–biotin-peroxidase complex (ABC) immunostaining technique was used. Slides were treated with 0.1% phenylhydrazine (Sigma Aldrich, St Louis, MO, USA) in phosphate buffered saline (PBS) containing 0.2% Triton X-100 (PBS/T) to block the endogenous peroxidase activity, and successively with 20% of normal goat serum (Vector Labs Inc., Burlingame, CA, USA) to minimize non-specific staining. Rabbit polyclonal antibodies against TRPV1 (Thermo Scientific, Waltham, MA, USA), diluted 1:500, and against CGRP (Chemicon, Temecula, CA, USA), diluted 1:1000, and a guinea-pig polyclonal antibody against SP (AbCam, Cambridge, UK), diluted 1:1200, were used as primary antibody. Biotin-conjugated goat anti-rabbit and anti-guinea-pig sera (Vector), both diluted 1:400, were used as secondary antiserum. The immunoreaction was revealed with 30 min of incubation with the ABC (BioSpa Div. Milan, Italy), diluted 1:250, and followed by incubation with a solution of 0.1 M phosphate buffer (PB), pH 7.3, containing 0.05% 3, 3′-diaminobenzidine (Sigma Aldrich, St Louis, MO, USA), 0.04% nickel ammonium sulfate and 0.01% hydrogen peroxide. All antisera and ABC were diluted in PBS/T. The specificity of the TRPV1 antibody has been validated by Western blot analysis on protein samples of human pre-term and adult TG and caudal medulla oblongata, and reported in a previous work [30]. Negative control preparations were obtained either by incubating tissue sections with the diluted primary antibody preabsorbed with 10 mM of the respective peptide for 24 h at 4 °C or by omitting the primary antibody. Cresyl violet, Black-Gold II staining kit (Biosensis, Thebarton, Australia) and/or Klüver–Barrera techniques were used as Nissl and myelin stainings. Observations and photographs were made with a photomicroscope Olympus BX61 (Hamburg, Germany), and with a slide scanner Nanozoomer 2.0-RS (Hamamatsu).

### 2.3. Morphometric Analysis

Morphometric analysis was performed on cuneate nucleus, gracile nucleus, external cuneate nucleus, Locus K, caudal spinal trigeminal nucleus substantia gelatinosa and magnocellular part, and solitary nucleus. Cell size analysis was performed on digital images captured with a 20× objective magnification. Cell mean diameters were automatically measured by Leica Application Suite Advanced Fluorescence (LAS AF) Software; statistical parameters (mean, minimum, maximum, S.D.) and histograms of the cell sizes were obtained by the Statistica 7 software (Version 7.0.61.0; StatSoft Inc., Palo Alto, CA, USA). Cell density (number of cells/mm^2^) was measured on digital images captured with a 10× objective magnification; statistical analysis was performed with One-way analysis of variance (ANOVA) and the Tukey’s *post-hoc* test by means of the software GraphPad Prism 6 for Windows (GraphPad Software, La Jolla, San Diego, CA, USA)

## 3. Results

**Figure 1:**
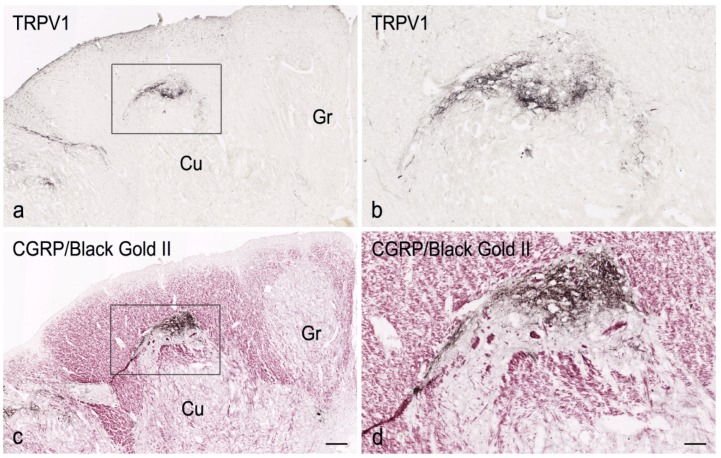
Full-term newborn, case 6. Left side dorsal quadrant in two consecutive sections of the caudal medulla oblongata immunostained for TRPV1 (**a**,**b**) and for CGRP followed by myelin Black Gold II counterstaining (**c**,**d**). Strongly TRPV1- and CGRP-like immunoreactive areas, along the dorsal border of the cuneate nucleus (Cu), are located in the Locus K (box in (**a**,**c**)) and are shown at higher magnification in (**b**,**d**), respectively. Gr, gracile nucleus. Scale bar: (**a**) = (**c**): 250 µm; (**b**) = (**d**): 50 µm.

**Figure 2:**
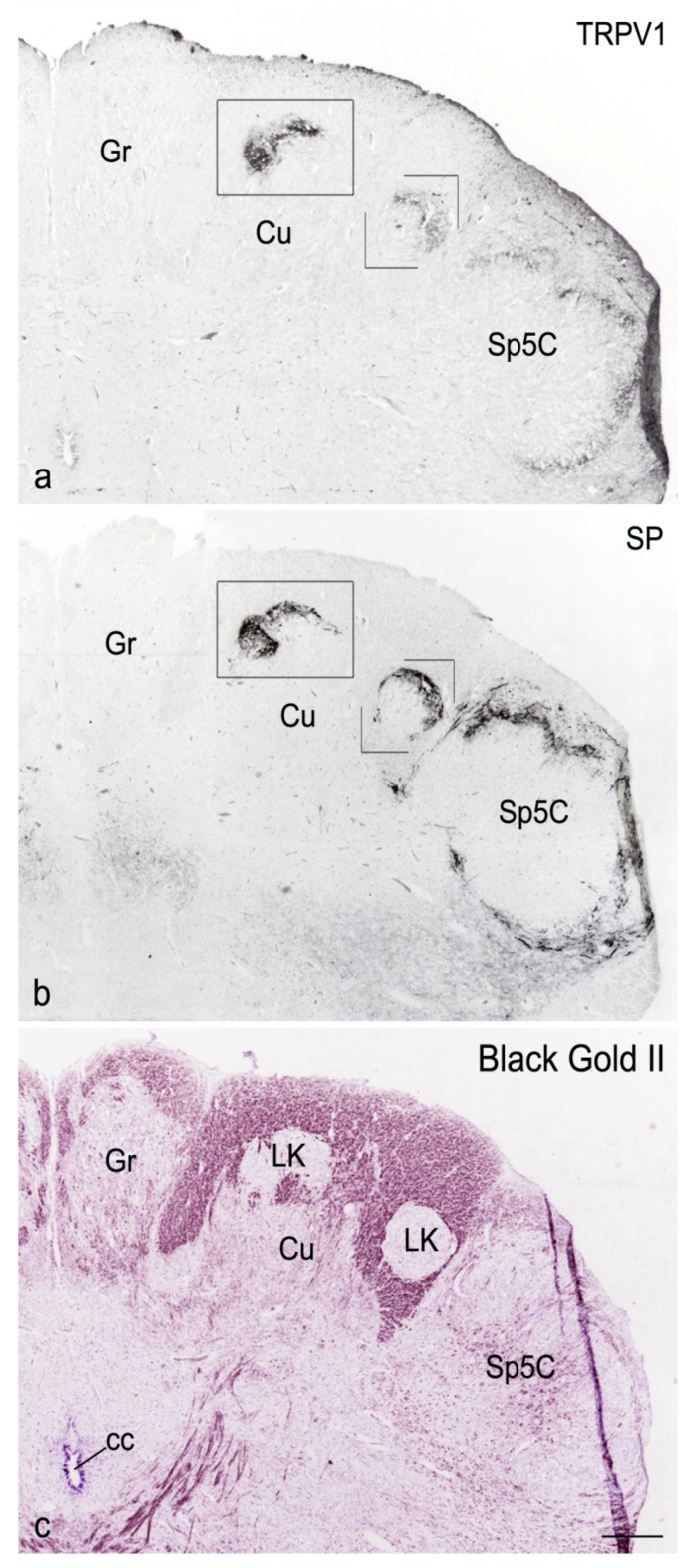
Full-term newborn, case 6. (**a**–**c**): right dorsal quadrant of three consecutive sections of the caudal medulla oblongata immunostained for TRPV1 (**a**) and SP (**b**), and stained for myelin with Black Gold II (**c**). TRPV1-LI (boxes in (**a**)) and SP-LI (boxes in (**b**)) are codistributed in a parallel way in the Locus K (LK) and in the spinal trigeminal nucleus (Sp5C) substantia gelatinosa. cc, central canal; Cu, cuneate nucleus; Gr, gracile nucleus. Scale bar: (**a**,**b**) = (**c**) 250 μm.

**Figure 3:**
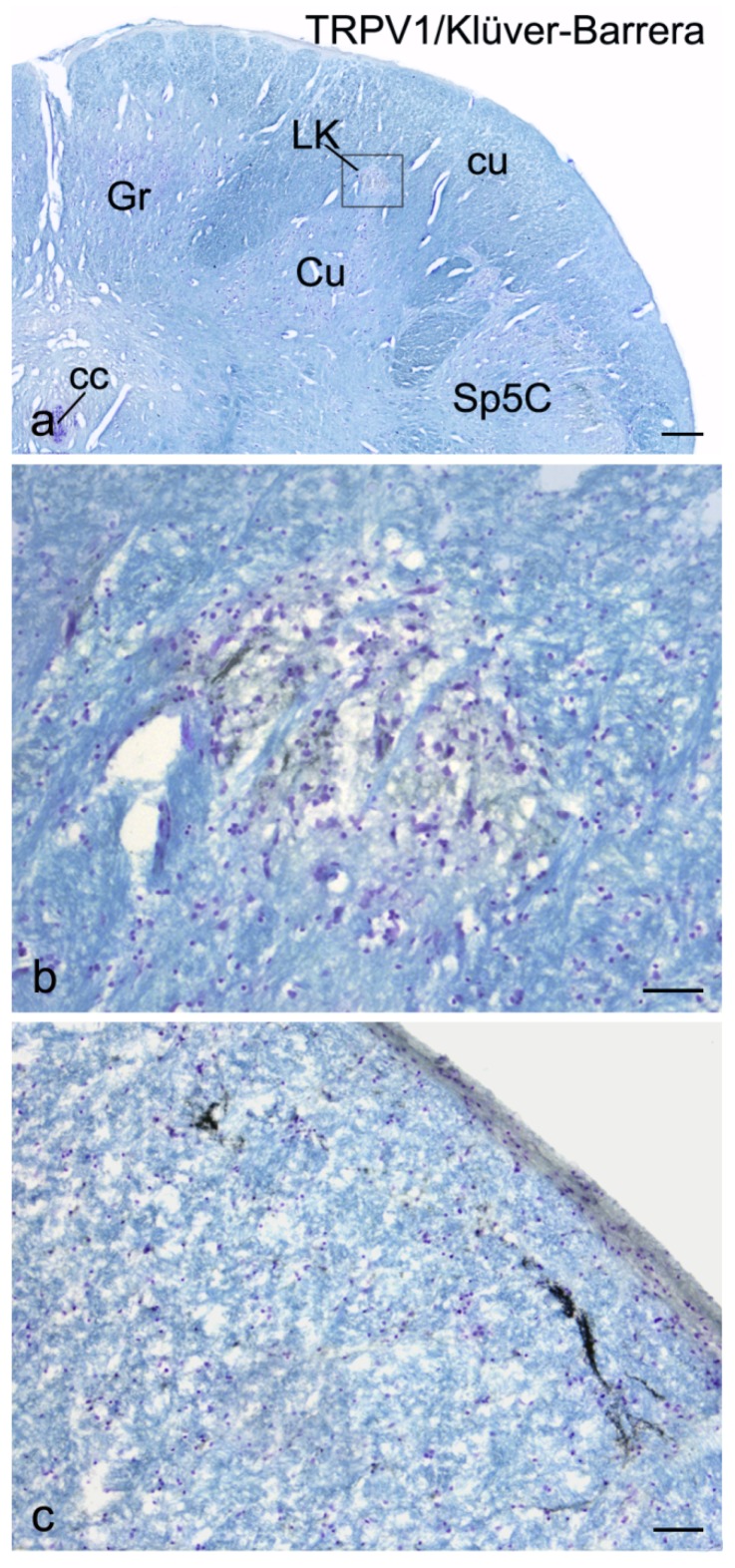
Adult, case 9. Right dorsal quadrant of caudal medulla oblongata immunostained for TRPV1 and counterstained with Klüver–Barrera (**a**). Locus K (LK) containing TRPV1-LI (box in (**a**)) is shown in (**b**) at higher magnification. **c**: thin fibre bundles in the dorsolateral fasciculus cuneatus (cu). cc, central canal; Cu, cuneate nucleus; Gr, gracile nucleus; Sp5C, caudal spinal trigeminal nucleus. Scale bar: (**a**) 250 μm; (**b**,**c**) 20 μm.

In the human caudal medulla oblongata of all examined specimens, from fetal and neonatal age (Figure 1a,b and Figure 2a) to adult life (Figure 3a,b), at levels between the pyramidal decussation and the obex, the territory of the cuneate nucleus contains distinct areas of gray matter that include TRPV1 strongly positive networks of varicose filaments and dot-like structures, interpreted as nerve fibres and terminals. By contrast, the remaining territory of the cuneate nucleus hosts scarce immunoreactivity. No evidence of immunoreactive cell bodies was found. Compared to the outcome in newborn tissue, the density of TRPV1-like immunoreactive structures appears reduced in adult specimens. No gender differences were observed. Immunostaining for CGRP and SP in adjacent sections allows for ascertaining that the TRPV1-LI is localized to the Locus K (Figure 1a–d and Figure 2a,b). However, though codistributed, the immunoreactivity for the receptor and the neuropeptides do not strictly overlap. In fact, though present in the superficial dorsal edge of the gray area, the bulk of TRPV1-LI, compared with the CGRP- and SP-LI, occupies a deeper zone of the Locus K (Figure 1a–d and Figure 2a,b). This is congruent with the previously described localization of the three markers in the substantia gelatinosa of the caudal spinal trigeminal nucleus [27]. As described for the neuropeptides [67,68,89], TRPV1-like immunoreactive fibres, either isolated or in thin bundles, are detectable within the cuneate fascicle (Figure 3c). No positive labelling is detectable at levels rostral to the obex. Alternate sections immunolabelled for TRPV1 and the neuropeptides, and histochemically stained for myelin (Figure 1 and Figure 2) effectively contribute to demonstrate the topographical localization of the TRPV1-LI of the Locus K and show its myeloarchitectural organization. In particular, analysis of myelin stained sections shows that the cuneate nucleus subregions with strong immunoreactivity to TRPV1 and the two neuropeptides contain rare myelinated fibres, whereas numerous stained fibres can be seen running across the territory of the main cuneate nucleus (Figure 1, Figure 2 and Figure 3). As previously described for the other markers, at certain levels, two distinct components of the Locus K, both containing TRPV1-LI, are detectable in the horizontal plane (Figure 2). The two regions are both located along the dorsal boundary of the cuneate nucleus, one in a dorsal and/or medial position, and the other one lateral to the cuneate nucleus and medial to the dorsomedial end of the caudal spinal trigeminal nucleus substantia gelatinosa (Figure 2). The medially located region may show a triangular, oval or arched profile, whereas the lateral one is round-shaped and the immunoreactivity is mostly confined to its crescent-shaped dorsal border (Figure 2). The histochemical stainings show that myelinated fibres, abundant in the cuneate nucleus, gracile nucleus, and caudal spinal trigeminal nucleus magnocellular part, conversely are scarce in both the Locus K and caudal spinal trigeminal nucleus substantia gelatinosa (Figure 1c,d, Figure 2c and Figure 3a,b).

**Figure 4:**
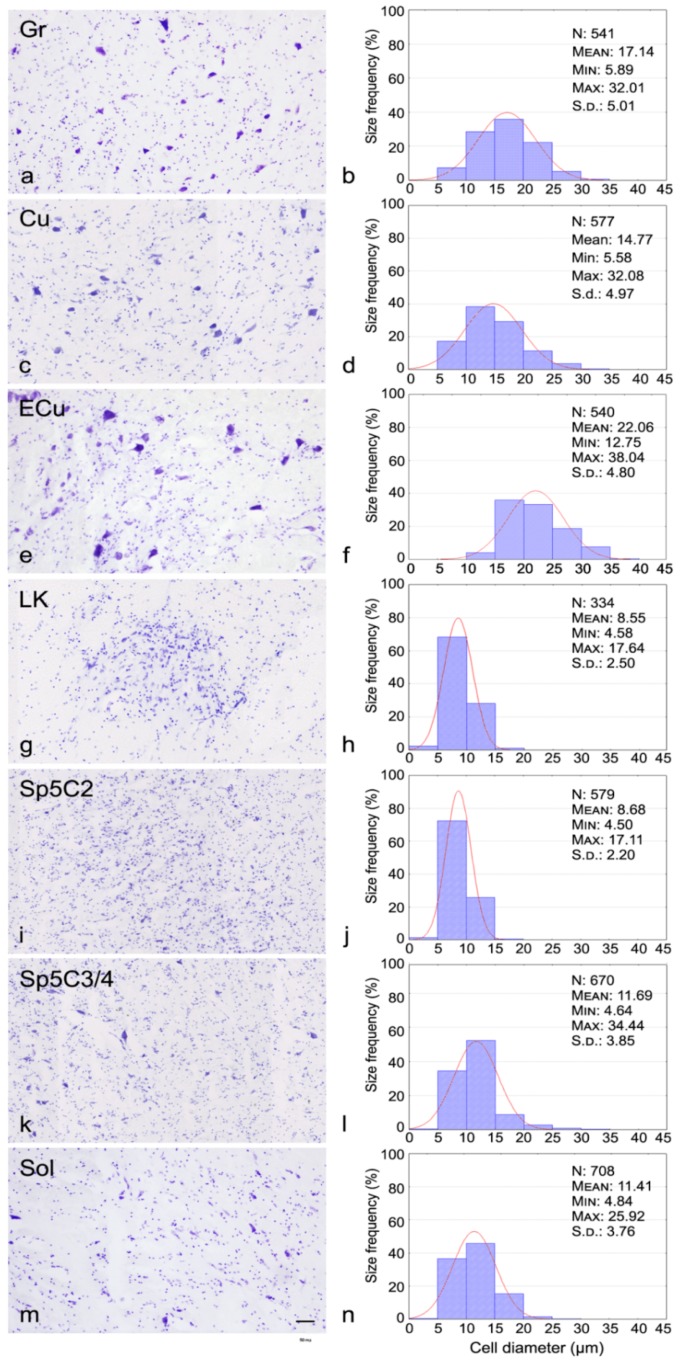
Adult, case 9. Nissl stained gracile nucleus (Gr), cuneate nucleus (Cu), external cuneate nucleus (ECu), Locus K (LK), caudal spinal trigeminal nucleus substantia gelatinosa (Sp5C2) and magnocellular region (Sp5C3/4), solitary nucleus (Sol) and relative size frequency histograms. In histograms, *x*-axis values represent the mean cell diameters expressed in μm, *y*-axis values report the relative percent frequency. Curves superimposed on the histograms represent the theoretical normal distribution. Scale bar: (**a**, **c**, **e**, **g**, **i**, **k**) = (**m**) 50 μm.

**Figure 5:**
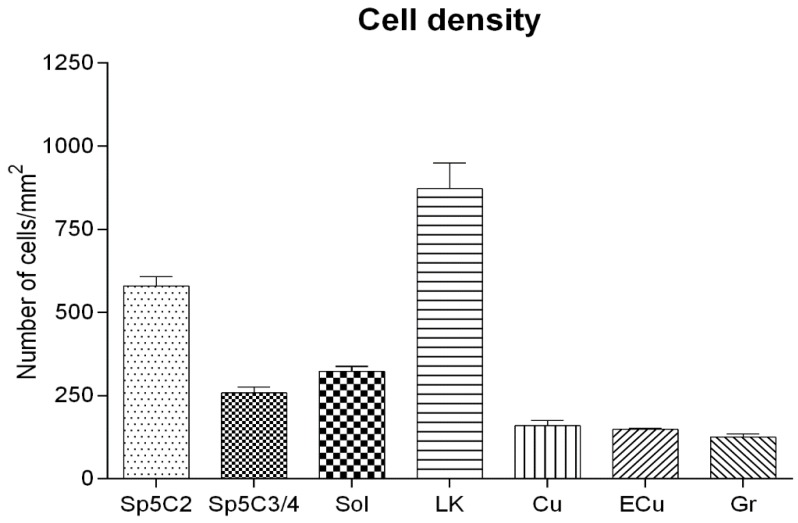
Adult, case 9. Histogram of mean cell density in the Locus K (LK) as compared to protopathic sensory nuclei and dorsal column nuclei of the human medulla oblongata. Differences in density among the seven regions are statistically significant (see Table 2). Caudal spinal trigeminal nucleus substantia gelatinosa (Sp5C2) and magnocellular region (Sp5C3/4), solitary nucleus (Sol), cuneate nucleus (Cu), external cuneate nucleus (ECu) and gracile nucleus (Gr).

Nissl staining performed on adult tissue sections (Figure 4) indicates that, in the LK, the cells are smaller and more closely packed (Figure 4g,h and Figure 5) than in the cuneate nucleus (Figure 4e,f and Figure 5), the histological aspect of the Locus K appearing rather similar to that of the caudal spinal trigeminal nucleus substantia gelatinosa (Figure 4i,j and Figure 5). The obvious tissue structure differences are proved by the analysis of cell size (histograms in Figure 4) and density (Figure 5). In the Locus K, as in the trigeminal substantia gelatinosa, the measured neurons show mean cell diameters between 5 and 18 µm (mean 8.55 and 8.68 µm, respectively), whereas, in the magnocellular part of the caudal spinal trigeminal nucleus, the mean diameters range 5 to 35 µm (mean 11.7 µm) and in the solitary nucleus 5 to 26 µm (mean 11.4 µm). In dorsal column nuclei, namely cuneate, external cuneate and gracile nuclei, the cell size is definitely larger, the mean cell diameter ranging 5 to 32 µm (mean 15 µm) in the cuneate and gracile nuclei, and 13 to 38 µm (mean 22 µm) in the external cuneate nucleus. As for the cell density (Figure 5), the mean value is 872.37/mm^2^ in Locus K, 579.63/mm^2^ in the substantia gelatinosa of caudal spinal trigeminal nucleus, 257.7/mm^2^ in the magnocellular part of caudal spinal trigeminal nucleus, 323.09/mm^2^ in the solitary nucleus, and between 125 and 159/mm^2^ in the dorsal column nuclei (cuneate, external cuneate and gracile nuclei). One-way ANOVA showed that scored differences in density are statistically significant (*p* < 0.0001); single *p*-values adjusted for multiple comparisons among the seven examined nuclear regions are reported in Table 2.

## 4. Discussion

The results obtained provide evidence for the presence of TRPV1-LI, in the form of a network of nerve fibres and terminals, within a set of distinct subnuclear areas located in the territory of the human cuneate nucleus, which we designate as Locus Kalaris or, briefly, Locus K. In a previous study, we provided a three-dimensional reconstruction of those areas [92], showing that the Locus K spans longitudinally from the pyramidal decussation to the obex level, first appearing caudally at the level of the cuneate nucleus caudal pole, and being located along the dorsal border of the cuneate nucleus. Similarly to several other markers, such as neuropeptides and molecules indicative of trophism and neuroplasticity [70,71,92,93,94,95,96,97], TRPV1-LI is detectable in the Locus K throughout life, from fetal to adult age. The present study also provides the first description of the morphometric features of the Locus K and a comparative analysis between Locus K and a number of the human medulla oblongata sensory nuclei. Among them, the mean cell size shows the lowest values in the Locus K and in the spinal trigeminal nucleus substantia gelatinosa, is somewhat higher in the spinal trigeminal nucleus magnocellular part and solitary nucleus, and is by far larger in the cuneate, gracile and external cuneate nuclei. A similar, though reversed, trend among the same nuclei is maintained with regard to the mean cell density, which shows the highest value in the Locus K followed by the spinal trigeminal nucleus substantia gelatinosa, whereas it lessens in the spinal trigeminal nucleus magnocellular part and solitary nucleus, and is greatly reduced in the cuneate, external cuneate and gracile nuclei. Thus, the concurrent immunohistochemical labelling for TRPV1 and the neuropeptides CGRP and SP (this study), which in turn are codistributed with several other markers [92], and the outcome of the cyto- and myeloarchitectural analysis, display the remarkable similarity in the neurochemical and structural arrangement between the Locus K and the protopathic sensory nuclei of the human medulla oblongata. Moreover, the pattern of immunolabeling and relative distribution of TRPV1-, CGRP- and SP-LI uphold the possibility that the Locus K is structurally organized in a laminar pattern, likewise the spinal trigeminal nucleus substantia gelatinosa. All of these observations induce consideration of a role in protopathic sensory neurotransmission for the Locus K and a functional involvement of TRPV1 in it, similar to that proposed in the spinal dorsal horn and, more generally, in the protopathic sensory nuclei. Ample evidence on the neurochemical anatomy of the somatosensory system in different animal species, including man, shows that immunoreactivity to several neuropeptides is concentrated in the superficial laminae of the spinal dorsal horn, in the caudal spinal trigeminal nucleus substantia gelatinosa, and in the solitary nucleus, being generally scarce to absent in the dorsal column nuclei [92] (and references therein), [100,101]. In a similar way, at a central level, the majority of TRPV1-containing structures occur in the superficial laminae of the rat spinal cord dorsal horn [22,102,103,104,105], in the rat [106] and human trigeminal substantia gelatinosa [27], and have a recognized role in transduction and transmission of noxious stimuli. In these territories, TRPV1 has been localized to unmyelinated (C) or thinly myelinated (Adelta) primary sensory afferents [1,11,14,102,106] that terminate mostly in lamina I and the inner part of lamina II of the rat spinal cord dorsal horn [102,103]. We showed a similar distribution in the human spinal trigeminal nucleus substantia gelatinosa [30]. However, postsynaptic TRPV1 has also been reported in the rodent superficial dorsal horn [103,107,108,109]. Moreover, together with that in somatic pain perception, TRPV1 appears to play an important role in visceral pain. In fact, at L4-S1 levels of the spinal cord dorsal horn [104,110] and sacral dorsal commissural nucleus [111], TRPV1 expression has been associated with visceral afferents innervating the urinary bladder and other pelvic organs, and TRPV1-bearing terminals have been shown in the solitary nucleus [112,113].

The existence of a region with the neurochemical and structural characteristics as the Locus K in the territory of the dorsal column nuclei, as well as the occurrence of TRPV1-LI in it, may sound in marked contrast with the classical functional role of those nuclei, which is epicritic sensibility. However, in keeping with a role in pain and protopathic perception, the dorsal columns, largely composed of thick myelinated fibres involved in the transmission of fine touch, vibratory sense and proprioception, also include a large proportion of thin and unmyelinated fibres [114,115,116], which may reach up to 25% in the human sacral spinal cord [117]. In the rat, these fibres have been identified as primary afferents and many of them are immunoreactive to CGRP and SP [117,118,119,120,121]. Additionally, nociceptive second order sensory fibres run in the dorsal column, composing the post-synaptic dorsal column (PSDC) pathway. The latter originates from neurons located in the central area of the spinal cord [122,123,124] and includes neuropeptidergic fibres [125]. The PSDC pathway carries visceral nociceptive information and clinical reports show that its surgical interruption effectively relieves intractable visceral pain in cancer patients [124,126,127]. Thus, on the one hand, the main involvement in sensory neurotransmission for TRPV1 and several neuropeptides, namely CGRP and SP, remains related to the protopathic sensory perception. On the other hand, the possibility that these molecules play a role in the epicritic sensibility classically attributed to the dorsal column nuclei may also be taken into account. In fact, at both peripheral and central level, TRPV1 may also contribute to mechanotransmission, especially after injury [108,128,129,130,131], and TRPV1-immunostaining has been detected in laminae III-V of the spinal cord dorsal horn, receiving, among others, primary afferents involved in proprioception [104]. Moreover, TRPV1- [132] and CGRP-positive fibres [128] have been shown to innervate light touch mechanoreceptor Meissner’s corpuscles in monkey and rat, respectively, and SP-positive fibres also occur in human and rat Meissner’s corpuscles and other mechanoreceptors [133,134,135]. We did not detect TRPV1-positive cell bodies in the LK. However, besides the likely prospect that the TRPV1-like immunoreactive fibres of the LK belong to neurons composing a sensory pathway, the possibility that, at least in part, they represent processes of local neurons or glial cells can not be ruled out, as shown in the rat spinal cord, with a role for the receptor in the control of pain transmission and onset of neuropathic pain disfunctions, such as hyperalgesia and allodynia [22,109,107]. Finally, the possibility should also be considered that the TRPV1-like immunoreactive fibres in the LK belong, at least in part, to descending projections. In fact, experimental evidence underlines the role of supraspinal TRPV1 in pain modulation, the rostral-ventrolateral medulla (RVM), periaqueductal grey (PAG), amygdala, solitary tract nucleus, locus coeruleus, somatosensory and anterior cingulated cortex, and insula being the territories most involved in this functional meaning [15,136,137,138,139,140,141,142]. Although the knowledge of the TRPV1 role in most of these systems is still incomplete, the TRPV1-mediated activation of the PAG-RVM antinociceptive pain pathway has drawn attention as a possible pharmacological target for some types of intractable pain [142].

At the present time, the possible functional involvement of the Locus K remains a matter of speculation. The localization pattern of TRPV1-, CGRP- and SP-LI in it shows that the elements containing the three markers do not overlap perfectly. This agrees with our findings on the substantia gelatinosa of the spinal trigeminal nucleus [30] and suggests that, especially in the deep part of the Locus K, the TRPV1-LI may reside in non peptidergic [13,14] and perhaps in non presynaptic [102,143] elements. 

In conclusion, the Locus K is still a “Nucleus in Search of a Function”. However, on a positive note, TRPV1 must be considered one of the Characters playing in it.

## Figures and Tables

**Table 1:** List of specimens.

Case	Age	Sex	Primary Cause of Death	Post-Mortem Hours
1	Fetus 21 w.g.	F	Cardiorespiratory failure	29
2	Pre-term newborn 6 d (25 w.g.)	F	Pneumonitis	25
3	Pre-term newborn 1 d (34 w.g.)	M	Cardiorespiratory failure	29
4	Pre-term newborn (38 w.g.)	M	Cardiorespiratory failure	38
5	Full-term newborn (40 w.g.)	M	Cardiorespiratory failure	28
6	Full-term newborn 1 d	M	Cardiorespiratory failure	24
7	Full-term newborn 2 d	F	Persistence of fetal circulation	38
8	Full-term newborn 7 d	F	Cardiorespiratory failure	27
9	Adult 44 y	M	Stabbing	40
10	Adult 53 y	F	Cardiorespiratory failure	31
11	Adult 56 y	F	Cardiomyopathy	34
12	Adult 71 y	M	Renal failure	25

F, female; d, days; h, hours; M, male; y, years; w.g., weeks of gestation (calculated from the 1st day of the latest menstrual cycle).

**Table 2:** *P*-values, calculated by means of one way ANOVA followed by Tukey’s *post-hoc* test, relevant to pair-wise contrasts between mean cell densities of Locus K (LK) and those of protopathic sensory and dorsal column nuclei in the human medulla oblongata (see histogram in Figure 5). Each *p*-value is adjusted to account for multiple comparison (significance level: 0.05; confidence level: 95%). Caudal spinal trigeminal nucleus substantia gelatinosa (Sp5C2) and magnocellular region (Sp5C3/4), solitary nucleus (Sol), cuneate nucleus (Cu), external cuneate nucleus (ECu) and gracile nucleus (Gr).

Nuclei	Summary	Adjusted *p*-Value
Sp5C3/4 vs. Sol	*	0.0267
Sp5C3/4 vs. LK	**	0.0033
Sp5C2 vs. LK	****	<0.0001
Sp5C2 vs. Cu	ns	0.5456
Sp5C2 vs. ECu	****	<0.0001
Sp5C2 vs. Gr	ns	0.1186
Sp5C3/4 vs. Cu	****	<0.0001
Sp5C3/4 vs. ECu	****	<0.0001
Sp5C3/4 vs. Gr	****	<0.0001
Sol vs. LK	****	<0.0001
Sol vs. Cu	****	<0.0001
Sol vs. ECu	****	<0.0001
Sol vs. Gr	****	<0.0001
LK vs. Cu	****	<0.0001
LK vs. ECu	****	<0.0001
LK vs. Gr	****	<0.0001
Cu vs. ECu	ns	0.9998
Cu vs. Gr	ns	0.9203
ECu vs. Gr	ns	0.9771

*, *p* < 0.05; **, *p* < 0.005, ****, *p* < 0.0001; ns, not significant.
